# Prediction of Moisture and Aging Conditions of Oil-Immersed Cellulose Insulation Based on Fingerprints Database of Dielectric Modulus

**DOI:** 10.3390/polym12081722

**Published:** 2020-07-31

**Authors:** Yiyi Zhang, Sheng Li, Xianhao Fan, Jiefeng Liu, Jiaxi Li

**Affiliations:** Guangxi Key Laboratory of Power System Optimization and Energy Technology, Guangxi University, Nanning 530004, Guangxi, China; yiyizhang@gxu.edu.cn (Y.Z.); sheng_li@st.gxu.edu.cn (S.L.); xianhao_fan@163.com (X.F.); lijiaxi1995@st.gxu.edu.cn (J.L.)

**Keywords:** transformer, oil-immersed cellulose insulation, frequency-domain spectroscopy (FDS), aging condition, moisture evaluation

## Abstract

Frequency-domain spectroscopy (FDS) is demonstrated to be affected by electrode polarization and conductance behavior in the low-frequency ranges, which causes the unreliable prediction results of transformer cellulose insulation. In order to solve this issue, a fingerprint database based on the dielectric modulus is reported to predict the degree of polymerization (DP) and moisture content of cellulose insulation. In the current work, the relevant fingerprints that characterize the insulation conditions are obtained by studying the dielectric modulus curves of cellulose insulation with various insulation conditions, as well as the DC conductivity of transformer oil. Then, the dielectric modulus fingerprint database is established in the lab, and the accuracy of the reported fingerprint database is later verified. As a potential tool, the dielectric modulus fingerprint database is tested by several samples, and the results demonstrate that the accuracy of this method is more than 80%. In that respect, an interesting discovery of this paper is that the dielectric modulus fingerprint database may be a helpful tool for conditions prediction of the transformer cellulose insulation system.

## 1. Introduction

As key equipment in the electric power system, oil-immersed power transformers have made great progress in their electrical and mechanical properties; however, the cellulose insulation is still considered the weakest part of transformers [1,2,3,4,5]. In other words, the operating life of transformers mainly depends on the conditions of the cellulose insulation. In recent decades, conditions prediction and fault diagnosis of a transformer cellulose insulation system have been regarded as the interesting issues and attracted extensive attention [6,7,8,9].

The insulation conditions of transformer cellulose insulation will degrade with operation time, water, furfural, organic compounds, and other additional products. As a result, in the present research, the evaluation methods of oil-immersed cellulose insulation mainly include the degree of polymerization (DP), dissolved gas-in-oil analysis (DGA), furan analysis, and frequency-domain spectroscopy (FDS) technique [10,11,12,13]. Most of these methods and operations are believed to be tedious ways in the field detection process; in addition, the insulation performance of the equipment can be damaged by some mentioned methods. On the contrary, the dielectric response technique, which serves as one of the most predominant techniques in the insulation condition diagnosis field, is also classified into the time-domain response and the frequency-domain response. Compared to the frequency-domain response (FDR), the time-domain response (TDR) has been suggested to have several defects including poor anti-interference and high time-consumption; the FDR is, therefore, believed to be more interesting for researchers [8,14,15,16].

Reviewing the previous work, a typical approach based on FDR, which is realized by extracting the parameters from the frequency-domain spectroscopy (FDS) curves, is utilized for predicting the transformer insulation conditions [14,15,17]. In addition, the core opinion of such an approach is attempting to investigate the variation law of the relevant parameters versus insulation conditions, and the prediction of insulation conditions is, therefore, achieved [15]. Consulting existing studies, based on the continuous understanding of the effect mechanism of some factor (aging, moisture, temperature, etc.) on frequency dielectric response characteristics, many scholars indicate that the relevant feature parameters can be used to characterize the insulation conditions of the transformer, including complex permittivity ε*(*ω*), dielectric loss (*tanδ*), etc. [8,15,18]. 

However, from the review of previous research, most papers only focus on the influence of a single factor (aging, moisture, or temperature) on insulation performance [7,19,20,21]; thus, the comprehensive prediction of the aging conditions and moisture content of cellulose insulation is of greater interest to researchers [13,22]. Besides, one more important point is that the FDS curves are affected by electrode polarization and conductance in the low-frequency ranges [20]. Due to such a fact, the relaxation information will be obscured during the FDS test; thus, the FDS curves (including ε*(*ω*), *tanδ*, etc.) are no longer appropriate for researching the relaxation process in low-frequency ranges, which may lead to the failure of results.

Fortunately, the researcher suggests that the dielectric modulus M*(*ω*) can be regarded as an effective method to evaluate the insulation conditions without being affected by the mentioned electrode polarization [20]. Compared to the traditional measurement data of FDR, the relaxation response of the dielectric modulus can form an obvious “polarization peak” in the low-frequency range [23]. Consequently, the dielectric modulus can not only be used to highlight the relaxation information but also reduce or even overcome the impact generated by electrode polarization and conductance during the FDS test. Furthermore, the feature fingerprints of dielectric moduli represented by some typical parameters could be used to characterize the aging and moisture of the insulation material. Thus, these fingerprints can be considered a potential approach for conditions evaluation [15].

In conclusion, to avoid the influence generated by aging and the moisture effect on the prediction results of the insulation conditions, the fuzzy pattern recognition technique is applied to this goal under the help of feature fingerprints of the dielectric modulus. In the current work, 20 samples with various aging conditions and moisture contents are first prepared for extracting the mentioned feature fingerprints; then, the fingerprint database used for conditions prediction is established by using these fingerprints. The accuracy and feasibility of the established database are verified by the newly prepared samples. In that respect, an interesting discovery of this paper is that the dielectric modulus fingerprint database may be a helpful tool for conditions prediction of the transformer cellulose insulation system.

## 2. The Definition of the Frequency Dielectric Modulus

**Figure 1:**
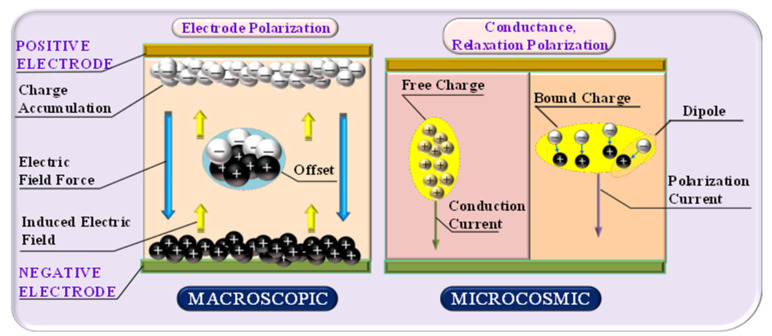
The frequency dielectric response process in the dielectric material.

In recent years, the dielectric response technique has been utilized in the conditions prediction of transformer cellulose insulation, where the frequency-domain spectroscopy (FDS) technique has attracted extensive attention from researchers [22,24]. The insulation information could be obtained from the dielectric material under an alternating electric field [25]. Specifically, the different behaviors of the relaxation polarization of transformer cellulose insulation with different moisture contents (*mc%*) and aging times can be observed in the FDS curves, which means that the FDS curve of the cellulose insulation is different under different insulation conditions [26]. However, relaxation polarization will be affected by other factors, including electrode polarization and conductance polarization. For further discussion, if the frequency is low enough (lower than 0.1 Hz), the charges near the electrode would be accumulated and have enough time to complete the electrode polarization process. Thus, unfortunately, an induced electric field, which is opposite to the direction of the original electric field, is established by these accumulated charges, and the original electric field is diminished due to this induced electric field, as shown in Figure 1.

From Figure 1, the relaxation process is obscured because of electrode polarization, which makes it very difficult to examine its behavior. As a result, the relaxation characteristics of FDS curves under the low-frequency region may be difficult to analyze: On the one hand, in low-frequency regions, the induced electric field is supposed to be in the opposite direction of the original electric field. On the other hand, the measured current generated in low-frequency regions is mainly formed by conductance current. In other words, the frequency dielectric response information in low-frequency regions is suggested to be the conductance rather than the relaxation polarization. To summarize, in the traditional frequency dielectric response, the information of conductance, rather than relaxation polarization, is extracted in the low-frequency regions, and the FDS curves would, therefore, no longer be suitable for evaluating the insulating conditions of cellulose [20].

Based on the limitations of FDS curves, the dielectric modulus is considered a powerful tool to overcome such defects. According to reference [23], the complex dielectric modulus M*(*ω*) can be defined as a reciprocal of complex permittivity, as shown in Equation (1).

(1) $$M^{*}(\omega )=\frac{1}{\varepsilon^{*}}$$

(2) $$\varepsilon^{*}(\omega )=\varepsilon^{\prime }-i\varepsilon^{\prime \prime }$$

where *ε′*(*ω*) and *ε″*(*ω*) are the real part and imaginary part of ε*(*ω*), respectively. As shown in Equation (3), the complex dielectric modulus M*(*ω*) can be expressed by *ε′*(*ω*) and *ε″*(*ω*).

(3) $$\begin{array}{c} M^{*}(\omega )=\frac{1}{\varepsilon^{*}}=\frac{\varepsilon^{\prime }}{\varepsilon^{{}^{\prime }2}+\varepsilon^{{}^{\prime }2}}+\frac{i\varepsilon^{\prime \prime }}{\varepsilon^{{}^{\prime }2}+\varepsilon^{{}^{\prime \prime }2}}\\ M^{\prime }(\omega )=\frac{\varepsilon^{\prime }}{\varepsilon^{{}^{\prime }2}+\varepsilon^{{}^{\prime \prime }2}}\\ M^{\prime \prime }(\omega )=\frac{\varepsilon^{\prime \prime }}{\varepsilon^{{}^{\prime }2}+\varepsilon^{{}^{\prime \prime }2}} \end{array}$$

As for the Debye relaxation, it is worth mentioning that the complex permittivity ε*(*ω*) can be expressed as Equations (4) and (5).

(4) $$\varepsilon^{*}(\omega )=\varepsilon_{\infty }+\frac{\varepsilon_{s}+\varepsilon_{\infty }}{1+{\left(\omega \tau \right)}^{2}}$$

(5) $$\left\{ \begin{array}{l} \varepsilon^{\prime }(\omega )=\varepsilon_{\infty }+\frac{\varepsilon_{s}-\varepsilon_{\infty }}{1+{\left(\omega \tau \right)}^{2}}\\ \varepsilon^{\prime \prime }(\omega )=\frac{(\varepsilon_{s}-\varepsilon_{\infty })\cdot \omega \tau }{1+{\left(\omega \tau \right)}^{2}} \end{array}\right.$$

where *ε_s_* is the static dielectric constant, *ε_∞_* is the dielectric constant when the angular velocity approaches infinity, *τ* is the relaxation time constant, and distribution factor *β* (0 < *β* < 1) is related to the FDS curve’s shape plotted in the complex plane [20].

(6) $$\begin{array}{ll} \frac{1}{\varepsilon^{*}(\omega )} & =\frac{1}{\varepsilon_{\infty }+\frac{\varepsilon_{s}+\varepsilon_{\infty }}{\left[1+{\left(i\omega \tau \right)}^{\beta }\right]}}=\frac{1}{\varepsilon_{\infty }}-\frac{1}{1+\frac{1+\varepsilon_{s}/\varepsilon_{\infty }}{\left[1+{\left(i\omega \tau \right)}^{\beta }\right]}}\\ & =\frac{1}{\varepsilon_{\infty }}-\frac{\frac{1}{\varepsilon_{\infty }}-\frac{1}{\varepsilon_{s}}}{1+{\left(i\omega \tau \right)}^{\beta }\cdot (\frac{\varepsilon_{\infty }}{\varepsilon_{s}})}=\frac{1}{M_{\infty }}-\frac{M_{\infty }-M_{s}}{1+{\left(i\omega \tau_{M}\right)}^{\beta }}=M^{*}(\omega ) \end{array}$$

where *M*_∞_ = 1/*ε_∞_*, *M*_s_ = 1/*ε*_s_, and *τ_M_* = *τ*(*ε_∞_/ε_s_*)^1/*β*^. Furthermore, *ε_∞_* is always smaller than *ε_s_*; therefore, it is easy to confirm that *τ_M_* is always smaller than *τ*. This indicates that, compared to the permittivity spectrum, the relaxation of the complex dielectric modulus moves to a higher-frequency region. Taking *M_∞_*, *M_s_*, and *τ_M_* into Equation (5), the real part and imaginary part expression of M*(*ω*) are obtained, as shown in Equation (7).

(7) $$\left\{ \begin{array}{l} M^{\prime }(\omega )=M_{\infty }+\frac{M_{s}-M_{\infty }}{1+{\left(\omega \tau_{M}\right)}^{2}}\\ M^{\prime \prime }(\omega )=\frac{(M_{s}-M_{\infty })\cdot \omega \tau_{M}}{1+{\left(\omega \tau_{M}\right)}^{2}} \end{array}\right.$$

In summary, the complex dielectric modulus M*(*ω*) can be regarded as the reciprocal of the complex permittivity ε*(*ω*) when the conductivity can be ignored [20]. In other words, as a frequency response characteristic parameter, M*(*ω*) is considered to be able to present the relaxation polarization information of the transformer cellulose insulation under an alternating electric field. Therefore, the M*(*ω*) is considered a powerful tool for conditions diagnosis of the transformer cellulose insulation systems.

## 3. The Frequency Dielectric Response Test and Analysis

### 3.1. The Frequency Dielectric Response Test

In this chapter, several groups of cellulose pressboards with different moisture contents (*mc%*) and aging conditions are utilized to investigate their frequency response characteristic under laboratory conditions. Subsequently, a series of experiments are carried out, and an oil-immersed cellulose pressboard with various insulation conditions is later prepared. Details of the cellulose pressboard and insulating oil are shown in Table 1.

**Figure 2:**
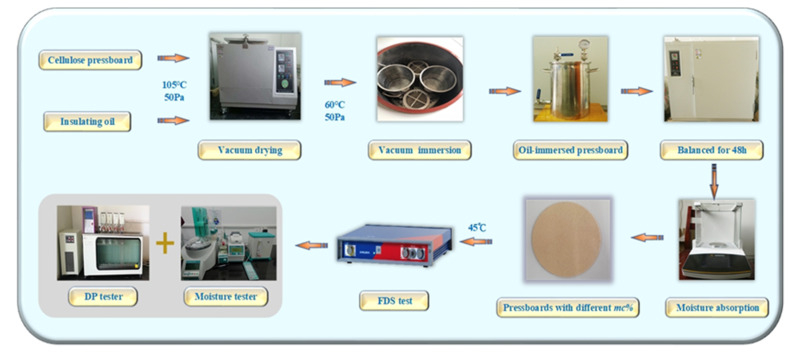
Experiment scheme of sample preparation and frequency-domain spectroscopy (FDS) test.

Details of the experimental process are shown in Figure 2. The drying and immersing of the pressboard are guaranteed to be carried out at 105 °C and 50 Pa, and 60 °C and 50 Pa, respectively, in lab conditions, and the time used for drying and immersing the samples is guaranteed to last 48 h. To accelerate thermal aging, the temperature of the aging box is set at 150 °C and a different aging time is selected; therefore, a pressboard with different aging conditions can be obtained. Afterward, each group of cellulose pressboards is placed in a precision electronic balance to absorb the expected moisture.

According to the standard IEC−60450, the DP test is carried out at 25 °C, lab conditions. The core method of the moisture test is Karl Fischer titration. According to the requirements of standard IEC−60814, the whole moisture test is completed in the laboratory conditions, 25 °C. Based on the principle of the frequency-domain dielectric response, the dielectric properties of the materials are measured under the AC electric field, in which the frequency range is 2 × 10^−4^−5 × 10^3^ Hz and the voltage amplitude is 200 V. With the help of the DIRANA/OMICRON, the FDS curves can be drawn, and the test is carried out under lab conditions with a constant temperature of 45 °C.

**Figure 3:**
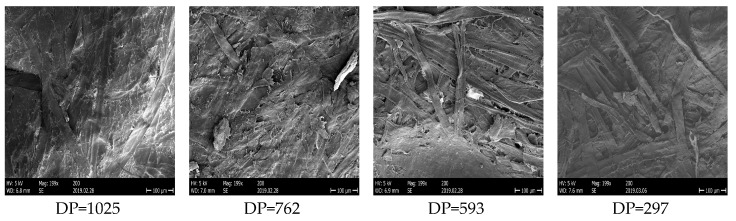
The SEM of cellulose pressboards with different DP values.

In order to consider the synergistic effect generated by aging and moisture on the predication results, 20 groups of cellulose pressboards with 5 kinds of aging conditions and 4 kinds of moisture content conditions are therefore prepared for discussion. The specific information is shown in Table 2. As shown in Figure 3, from the scanning electron microscope (SEM), the microscopic structure of cellulose can be changed with deeper aging conditions.

### 3.2. The Complex Permittivity Curves Analysis

**Figure 4:**
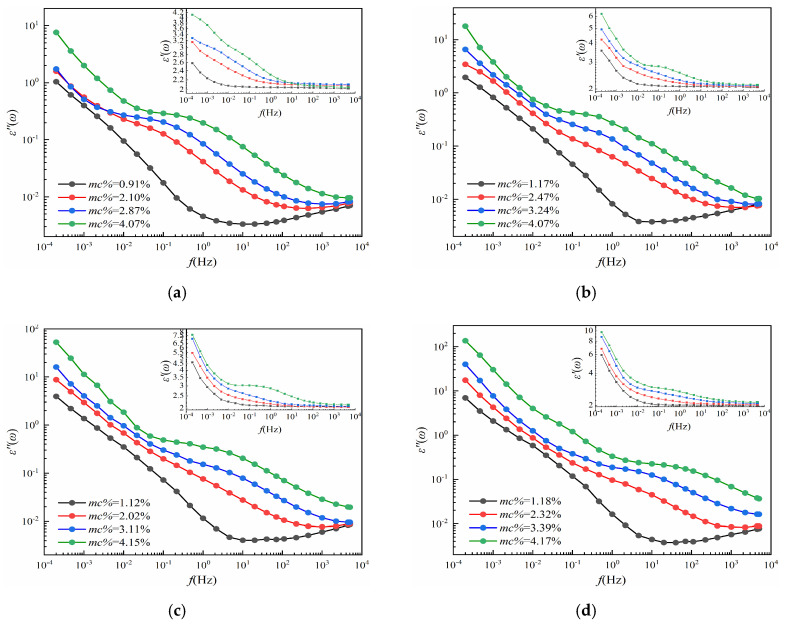

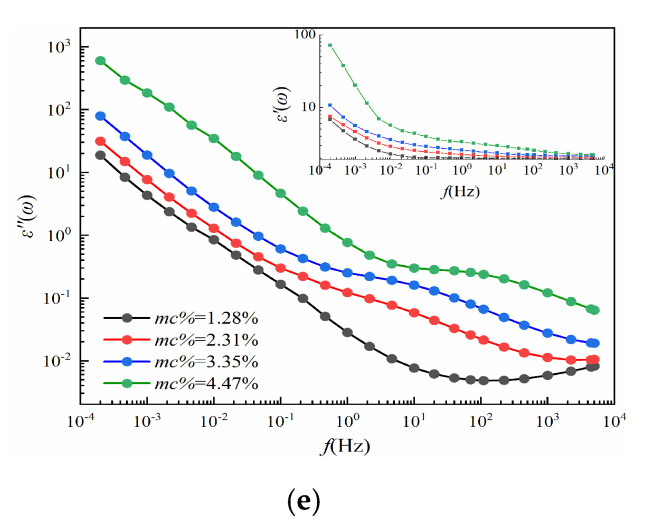
(**a**) The ε′(ω) and ε″(ω) curves of P_11_−P_14_; (**b**) the ε′(ω) and ε″(ω) curves of P_21_−P_24_; (**c**) the ε′(ω) and ε″(ω) curves of P_31_−P_34_; (**d**) the ε′(ω) and ε″(ω) curves of P_41_−P_44_; (**e**) the ε′(ω) and ε″(ω) curves of P_51_−P_54_.

It is worth mentioning that each group has the same aging time, therefore, the DP values of this group of cellulose pressboards are almost coincided and, thus, these pressboards are considered to keep the same aging conditions. Then, the frequency dielectric response test is launched in the three-electrode test cell under 45 °C by using DIRANA/OMICRON, and the *ε′*(*ω*) and *ε*″(*ω*) of cellulose pressboards with various *mc%* and aging times are drawn in Figure 4. In addition, some clear effects caused by moisture and aging are shown in Figure 4:

#### 3.2.1. Moisture Effects

Moisture content affects the whole frequency regions of ε″. The low-frequency region (10^−4^ Hz−10^−1^ Hz) of ε’ is also affected by moisture content, while little influence is on the high-frequency region (10^2^−10^4^ Hz) of ε’. Besides, with the increase in moisture content, the curves of *ε’* and *ε″* are considered to shift to the higher-frequency region, and the minimum value of ε″ gradually increases [8]. The reason caused by this phenomenon is that the water molecules are considered as the polar molecules, which means the increase in the number of water molecules leads to the increase in conductance and polarization loss. Moreover, it becomes easier for the oil−paper interface to accumulate the charges, leading to the interface polarization being established in a shorter response time. Therefore, the curves shift toward the higher-frequency region [27].

#### 3.2.2. Aging Effects

The complex permittivity, including *ε’* and *ε″*, in the low-frequency region is mainly affected by the values of DP, while the moisture content of the pressboard is low. Specifically, with the increase in aging time, the ε’ and ε″ in the low-frequency region gradually increase, while the high-frequency region almost remains unchanged. Such a phenomenon is caused by the water molecules and small acid molecules that are produced in the aging process of the pressboards. Then, the number of these strong polar molecules will increase, which leads to the interface being established in a shorter time. Similar to the moisture effect, the curves, therefore, shift toward a higher-frequency region.

#### 3.2.3. Summaries

**Figure 5:**
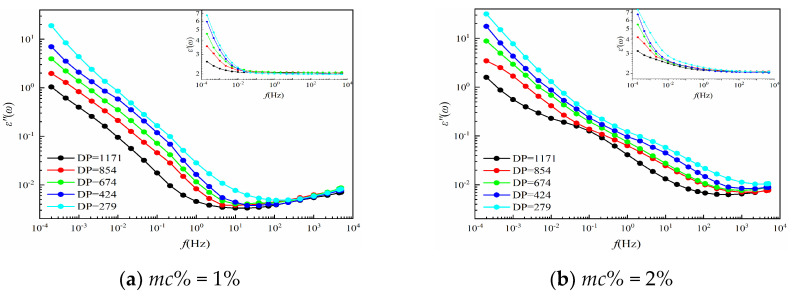

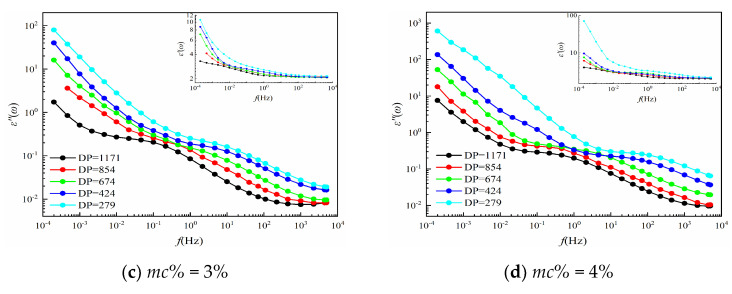
(**a**) The ε′(ω) and ε″(ω) curves of P_11_−P_51_; (**b**) the ε′(ω) and ε″(ω) curves of P_12_−P_52_; (**c**) the ε′(ω) and ε″(ω) curves of P_13_−P_53_; (**d**) the ε′(ω) and ε″(ω) curves of P_14_−P_54_.

From Figure 5, the ε″ curves no longer coincide in the high-frequency region. This phenomenon is similar to the moisture effect, which indicates that the influence of aging will be covered by the influence of moisture content on ε″. That is to say, if the moisture content is high enough, the performance of the cellulose pressboard will be dominated by moisture content [13]. That makes it difficult to distinguish the contribution of moisture and aging to the complex permittivity curves.

### 3.3. The Complex Dielectric Modulus Curves Analysis

**Figure 6:**
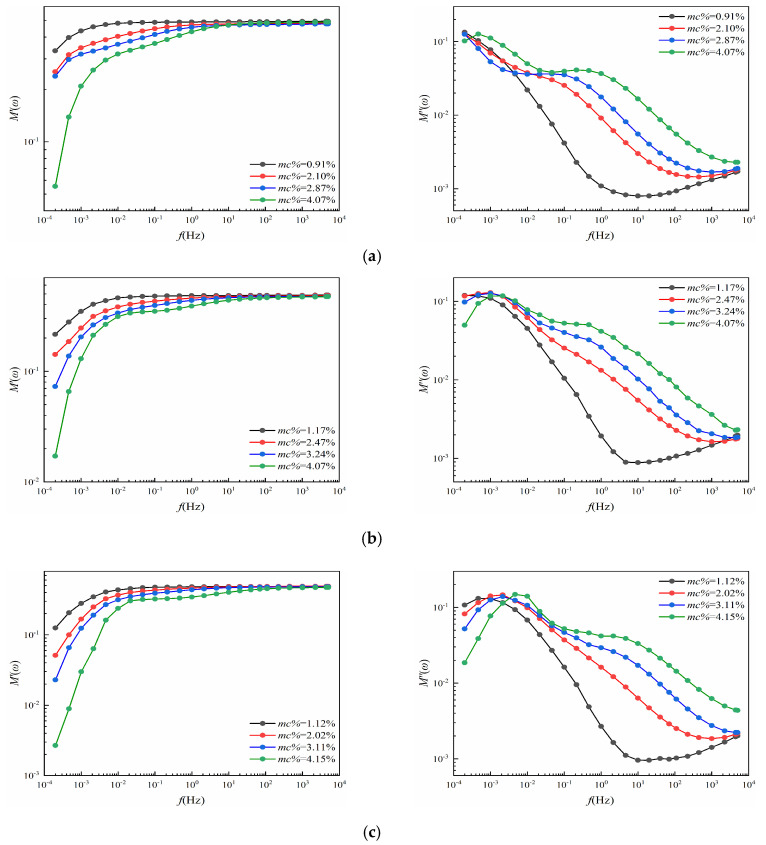

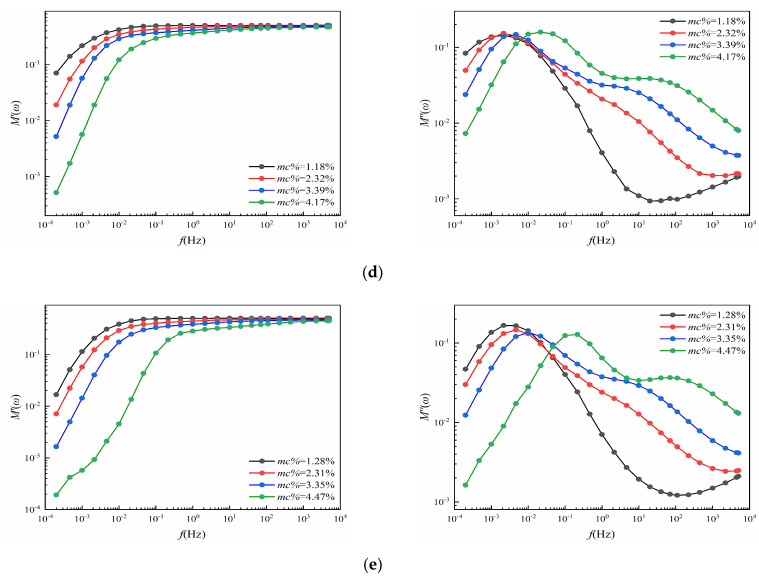
(**a**) The M′(ω) and M″(ω) curves of P_11_−P_14_; (**b**) the M′(ω) and M″(ω) curves of P_21_−P_24_; (**c**) the M′(ω) and M″(ω) curves of P_31_−P_34_; (**d**) the M′(ω) and M″(ω) curves of P_41_−P_44_; (**e**) the M′(ω) and M″(ω) curves of P_51_−P_54_.

According to Equation (3), as long as the ε*(*ω*) is determined, the M*(*ω*) can be calculated conveniently. Then, the curves of M*(*ω*) can be plotted in the *log*−*log* system, as is shown in Figure 6. 

Similar to Figure 4, Figure 6 is regarded as the contribution of the moisture effects and the aging effects.

#### 3.3.1. Moisture Effects

In the high-frequency region, the M’ curves from Figure 6 are regarded as a straight line overlapped by several curves, while, in the low-frequency region, the values of M’ negatively correlate with water content. Similar to the ε’, the M’ curves in Figure 6 are considered to shift to the high-frequency region with the increase in moisture content [23]. This phenomenon can also be explained by the increase in the number of water molecules, a kind of polar molecule.

Unlike the ε″ in Figure 4, several relaxation peaks are shown in the low-frequency region from the *M″* curves in Figure 6. One more interesting fact is that the position of the peaks on the curve is related to the water content. The relaxation peaks in the low-frequency region also gradually shift to a higher frequency with the increase in moisture content. The dielectric modulus is therefore considered as the potential tool to access more relaxation information.

#### 3.3.2. DP Effects

Similar to Figure 6, under similar moisture content conditions, the relaxation peaks in the low-frequency region also gradually shift to the higher-frequency region with a decrease in DP. This phenomenon, similar to the effects of aging on *ε″* in Figure 4, is still related to the polar molecules produced by aging.

#### 3.3.3. Summaries

Compared to Figure 6, the imaginary part of the complex permittivity of Figure 4 is negatively correlated with the frequency. Especially in the low-frequency regions, the curves of *ε*″(*ω*) dominated by conductance and electrode polarization are mainly too enormous in the low-frequency regions to distinguish its relaxation information [23]. However, from Figure 6, the relaxation peaks mainly caused by the relaxation polarization are presented in *M″*(*ω*) curves. The relaxation peak can be observed in the *M*″(*ω*) curves in the low-frequency regions, and the *M*″(*ω*) curves gradually shift to the high-frequency region with increasing *mc%* and aging time, in the full-frequency regions. In addition, the relaxation peaks are related to the feature of relaxation and, meanwhile, convey the rich insulation information of the dielectric response.

## 4. The Extraction of Fingerprint Parameters from the Dielectric Modulus

### 4.1. The Extraction of Fingerprints of S_1_, S_2_, and S_3_

In this chapter, several groups of data from the experimental results are proposed to form the feature fingerprints. The generally known rule is that the dielectric response characteristics of cellulose materials would change as their insulation conditions are altered [23]. As the paper reported [28], the polarization and conductance processes of the cellulose pressboard would be enhanced because the moisture molecules are regarded as a strong polar substance. In addition, the dielectric response characteristics of cellulose materials could be affected by the DP (degree of polymerization) value, which depends on its aging conditions. Therefore, to obtain more insulation information for characterizing the aging conditions and moisture content in a limited frequency band, the integral values in different frequency ranges can be regarded as a helpful tool to achieve this target, as shown in Equation (8).

(8) $$S_{i}=\int_{f_{1}}^{f_{2}}M^{*}(f)df(i=1,2,3,\ldots )$$

where *S_i_* is named the integral factor. As there is no clear definition for the range of the above frequency, a requirement is that the response curve in each frequency range should be sensitive to the change in insulation conditions. In addition, the integral values of the dielectric modulus in the different frequency region curves are selected as the fingerprints to eliminate the errors caused by a single frequency point. Therefore, in this work, three frequency ranges (10^−3^−10^−2^, 10^−1^−10^0^, 10^2^−10^3^ Hz) are chosen to calculate the integral values (*S*_1_, *S*_2_, and *S*_3_) to develop the fingerprint database. In addition, *S*_1_, *S*_2_, and *S*_3_ correspond to the regions of low frequency, medium frequency, and high frequency, respectively. These extraction formulas can be found in Equation (9), and the obtained results are as shown in Table 3.

(9) $$\left\{ \begin{array}{l} S_{1}=\int_{10^{-3}}^{10^{-2}}M^{\prime \prime }(f)df\\ S_{2}=\int_{10^{-1}}^{10^{0}}M^{\prime \prime }(f)df\\ S_{3}=\int_{10^{2}}^{10^{3}}M^{\prime \prime }(f)df \end{array}\right.$$

The influence mechanism of the single factor (such as aging time or *mc%*) on the dielectric modulus is discussed in previous research [5,20], but few of the works are focused on the synergistic effect of DP and *mc%*. In this part, the moisture, aging, and their combined effects on cellulose performance will be discussed.

With the increase in moisture content, it can reduce both the resistivity and mechanical strength of cellulose insulation [4], which more easily causes electrical breakdown. The dielectric loss of the pressboard also increases because of more water molecules, where it is easy to cause thermal breakdown. The hydrolysis of cellulose can also be caused by the moisture content of the pressboard, which breaks the molecular chain of cellulose and decreases its mechanical properties.

The decrease in DP value reduces the mechanical properties of cellulose, which becomes easier to damage. If the aging is serious (DP value is small enough), the cellulose will embrittle and fall off from the transformer windings. In addition, some chemicals in the reaction of cellulose, caused by aging, produce some water and organic acids; therefore, the molecular chain of cellulose will also experience breakdown [29], which also affects the properties of cellulose.

The effects of moisture and aging on the properties of cellulose are mutually promoted. On the one hand, the aging of insulation pressboards can produce some water molecules [4], which will affect the moisture content of the pressboards. On the other hand, the presence of water molecules in cellulose often leads to its hydrolysis, which produces some organic acid molecules, and the hydrolysis leads to the breakage of the cellulose chain, which affects the degree of polymerization directly [30]. In other words, the most reasonable method to predict the insulation condition is to consider the combined effects of aging and moisture on cellulose.

The S1, S2, and S3 in Table 3 are parameters without physical meanings. However, those integral factors’ values show the sensitivity to moisture content and aging conditions. For example, the values of the *S*_1_ change significantly with the increase in aging time. During the early aging and the mid aging of cellulose, the integral value grows with the increase in moisture content. While the pressboard is in the later period of aging, the integral value decreases with the increase in moisture. 

### 4.2. The Extraction of S_4_

As the published paper [24] suggested, although the cellulosic pressboard is under different insulation conditions, the shapes of the dielectric modulus spectrum may be similar because of the synergistic effect caused by *mc%* and aging activity. In addition, the performance of the DC conductivity of insulation oil *σ*(*T*) is reported to be sensitive to the change in insulation conditions [31], including aging and moisture. Therefore, to distinguish the contribution to the dielectric modulus spectrum caused by moisture and aging, the DC conductivity of insulation oil *σ*(*T*) is considered an available measure. To facilitate data processing, the values of *σ*(*T*) are expanded by a factor of 1 × 10^12^, and the obtained value is defined as *S*_4_.

Identical to *S*_1_−*S*_3_, from Table 4, the values of *S*_4_ are also considered to be related to the synergistic effect caused by *mc%* and aging. For the convenience and requirement of this work, the values of *S*_1_−*S*_4_ are tabulated in Table 5.

## 5. Feasibility Verification of the Fingerprint Database

### 5.1. The Introduction of Fuzzy Pattern Recognition

In this part, the method for the prediction insulation conditions of new samples by the dielectric modulus database is discussed. Referring to IEC 60422 and IEEE 62−1995 standards, the corresponding classification of the insulation conditions is given for predicting the transformer cellulose insulation system. Based on the comprehensive analysis of the above rules, the oil-immersed insulating pressboard is divided into 16 target states, as shown in Table 6.

The fingerprints (*S*_1_–*S*_3_) can be calculated according to Equation (9), while *S*_4_ can also be obtained by using the oil conductivity test. Thus, the values of *S_*1*_–S_*4*_* of new samples are obtained, with the help of relevant algorithms including normalization conversion (NC) and fuzzy pattern recognition (FPR), and the results of conditions (DP and *mc%*) are later computed.

Obviously, from Table 3, making the values of *S*_1_–*S*_3_ remain in the same data dimensions, the influence on evaluation results caused by the various dimensions can, therefore, be eliminated. The NC equation, shown in Equation (10), is utilized for eliminating such influence.

(10) $${x^{\prime }}_{ij}=\frac{x_{ij}-\min\left\{x_{ij}\right\}}{\max\left\{x_{ij}\right\}-\min\left\{x_{ij}\right\}}$$

where the values of *x_ij_’* are transformed data and the values of *x_ij_* are data before the transform. In this work, *i* represents the amount of target state, *i* ∈ (1, 20). Meanwhile, *j* represents the numbers of fingerprints contained in each target state, *j* ∈ (1, 4). The max{*x_ij_*} is the maximum value of the *j-th* column in the fingerprint database and min{*x_ij_*} is the minimum value.

The fuzzy pattern recognition algorithm is intended to predict the conditions of the cellulose pressboard. At first, the obtained standard target state (*TS*) shown in Table 5, as well as the unknown samples (*X*), are studied by the computers, automatically. Afterward, pattern recognition is operated, which is realized by comparing the Euclidean distance *d(X, TS)* between the *X* and *TS*. In addition, the close degree *N(X, TS)* is regarded as a helpful tool to achieve such recognition. The relationship between *d(X, TS)* and *TS* can be expressed by Equation (11).

(11) d(X,TS)=1−N(X,TS)

*N(X, TS)* can be calculated by Equation (12), where *n* represents the number of fingerprint parameters contained in each *TS*, *n* = 4. *X*(*i*) and *TS*(*i*) are the *i-th* fingerprints of the *X* and *TS*, respectively.

(12) $$N(X,TS)=1-\frac{1}{\sqrt{n}}\sqrt{\sum_{i=1}^{n}\left[NS(i)-S(i\right){]}^{2}}$$

**Figure 7:**
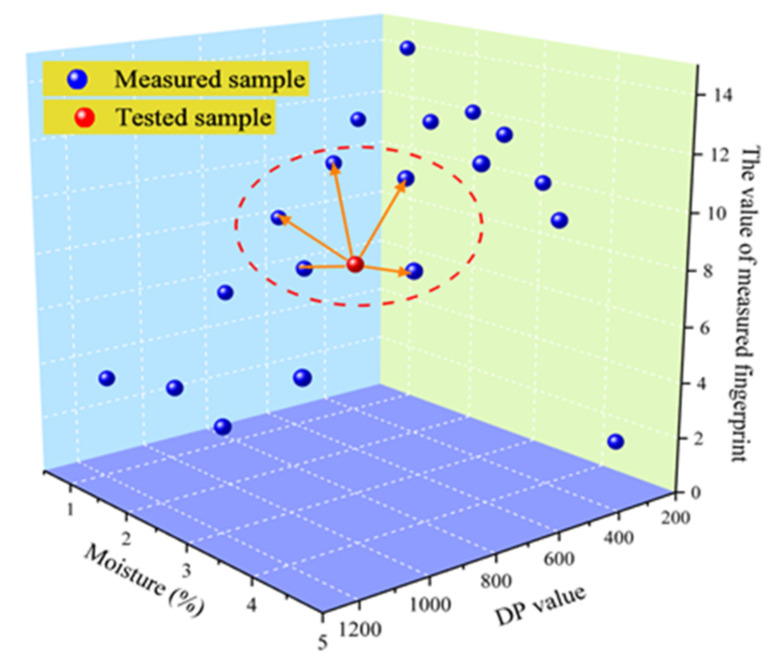
Principle diagram of fuzzy recognition.

In other words, the recognition algorithm refers to the comparison of the fingerprint characteristics of the tested samples and the fingerprint database to find the corresponding or closest target state. The principle diagram of fuzzy recognition is shown in Figure 7.

### 5.2. Identification of New Samples

**Figure 8:**
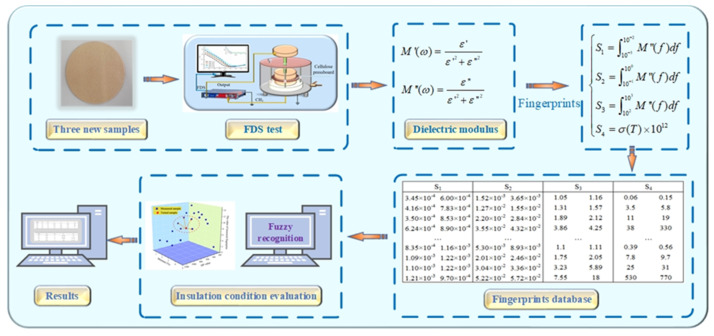
The feasibility verification of the fingerprint database.

**Figure 9:**
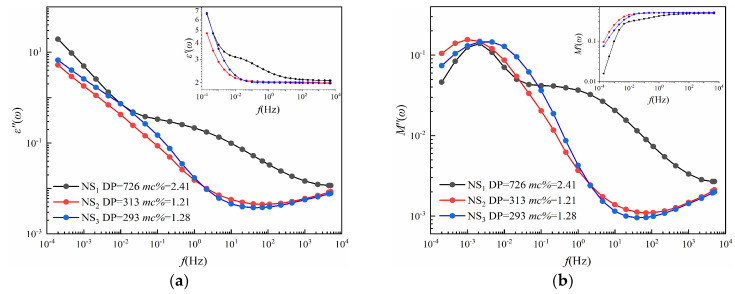
(**a**) *ε’*(*ω*) and *ε″*(*ω*) of *NS*_1_, *NS_2_,* and *NS*_3_. (**b)** *M’*(*ω*) and *M″*(*ω*) of *NS*_1_, *NS_2_,* and *NS*_3_.

In this chapter, the insulation conditions of three new samples (*NS*_1_−*NS*_3_) are predicted according to Figure 8. As shown in Figure 2, the preparation of new cellulose samples has the same process, and the results of the FDS test are shown in Figure 9. Details of the new pressboard, including their insulation conditions and measured fingerprints (*S_*1*_–S_*4*_*), are shown in Table 7.

As shown in Table 7, the moisture content and DP value of NS_2_ (DP = 313, *mc%* = 1.21) and NS_3_ (DP = 293, *mc%* = 1.28) are very close, which are also reflected in their curves in Figure 9. However, their dielectric modulus fingerprint parameters are different. Although the values of *S*_1_ (NS_2_ = 1.06 × 10^−3^, NS_3_ = 1.26 × 10^−3^) and *S*_3_ (NS_2_ = 1.16, NS_3_ = 1.11) are relatively close, the values of *S*_2_ (NS_2_ = 6.7 × 10^−3^, NS_3_ = 1.00 × 10^−2^) and *S*_4_ (NS_2_ = 0.46, NS_3_ = 1.10) are quite different, which means that we have the criteria to distinguish those two conditions.

The closeness between the three new samples and the fingerprint database after fuzzy identification, as well as the corresponding degree of polymerization and moisture content, are shown in Table 8.

For NS_1_, the predictive condition of the database is T_8_ (DP = 700−900, *mc%* > 4%, Serious damp), and the prediction of DP values is correct (practical DP = 726). However, the practical moisture content of NS_1_ is 2.41, which should be considered as slight damp. These results indicated that the error may be caused by the moisture identification or other factor; future research will focus on this problem. Besides, the accuracy of the proposed method is also related to the number of reference samples (in this work, the number of reference samples is 20). With the increase in the number of samples, the accuracy will improve.

As for NS_2_ and NS_3_, the condition suggestions given by the database are T_13_ (DP = 500−700, *mc%* = 0%−1.5%, Dry) and T_17_ (DP < 300, *mc%* = 0%−1.5%, Dry), respectively, while the practical conditions of NS_2_ and NS_3_ are DP = 313 and *mc%* = 1.21, and DP = 293 and *mc%* = 1.28, respectively. This means that the accurate prediction results can be given by the dielectric modulus fingerprint database. In conclusion, although the individual error of the result is found, the fingerprint database is still considered a potential tool for cellulose insulation prediction.

### 5.3. Comparison with Grey Relational Analysis

Among the methods that have been proposed, as the grey relational analysis method can evaluate multiple conditions at the same time, this method is chosen to compare to the method proposed in this paper. The core idea of the grey relational analysis is the correlation degree between the condition data (in this paper, *S*_1_–*S*_4_ of new samples are regarded as the condition data) and the reference data (*S*_1_–*S*_4_ of papered pressboard in Table 5 are regarded as the reference data). The greater the correlation degree, the closer the condition data are to the reference data, and the insulation conditions of the pressboards are, therefore, evaluated according to that.

According to the grey relational analysis, an algorithm is established, and the insulation conditions prediction of NS_1_, NS_2_, and NS_3_ will be carried out. The results are shown in Table 9. From Table 9, the predictive condition of NS_1_ is T_4_ (DP = 900−1400, *mc%* >4%, Serious damp), and compared to its practical conditions, the practical DP is 726; however, the predictive DP is more than 900. Unfortunately, significant errors are also between the predictive *mc%* (>4%) and practical mc% (= 2.41). As for NS_2_, its predictive condition is T_17_ (DP < 300, *mc%* = 0%−1.5%, Dry), and the practical moisture content of NS_2_ is 1.21, which means the right prediction results are given by the grey relational analysis. However, some errors still exist between the predictive DP (DP < 300) and practical DP (DP = 313). From the results of NS_3_, similar to the NS_2_’s results, some errors also still exist between the predictive DP (DP = 300−500) and practical DP (DP = 293).

In conclusion, compared to the grey relational analysis, the dielectric modulus fingerprint database is demonstrated to be more accurate. With the increase in the number of prepared pressboards, the accuracy of the dielectric modulus fingerprint database will be further improved.

## 6. Conclusions

Compared to the traditional methods, such as the Karl Fischer titration (KFT) and viscosity method, the dielectric modulus fingerprint database cannot destroy the cellulose pressboard. In addition, compared to the FDS methods, the proposed method can predict the insulation conditions of the cellulose pressboard when including both the aging effect and moisture effect. However, the proposed method is an off-line technique, which means this method cannot predict the insulation conditions of the running transformers. In addition, the established model depends on the used materials, which might limit the applications in the field conditions. Further optimization and modification are thus needed to overcome this issue.

The dielectric modulus can not only be used to highlight the relaxation information but also reduce or even overcome the impact generated by electrode polarization behavior and conductance behavior during the FDS test. This feature allows the dielectric modulus to be a potential tool for analyzing and evaluating the insulation conditions of transformer cellulose insulation. Meanwhile, the dielectric modulus fingerprint database is established, and the synergistic effect of moisture and DP can be verified. Although there are some errors in the results, this database can still be considered an interesting approach to evaluate the insulation conditions of transformers. The present analysis and contributions have led to the following conclusions.

- As a frequency-response characteristic parameter, M*(*ω*) is certificated to be able to present the relaxation polarization information of the transformer cellulose insulation in the course of the FDS test.
- It is found that the imaginary part of the dielectric modulus could form an obvious relaxation peak in the low-frequency regions, which could be utilized to extract the feature fingerprints to characterize the aging and moisture of cellulose insulation by using integral operation.
- The synergistic effect generated by moisture and aging can be separated or distinguished by using DC conductivity. The novelty of this work is in an exploration of the dielectric modulus as a useful tool to extract the parameters to build a database for the comprehensive prediction of aging and moisture.
- It is proved that the reported feature fingerprint database could serve as a potential tool for the comprehensive prediction of transformer cellulose insulation.

## Figures and Tables

**Table 1:** Fundamental parameters of both cellulose pressboard and oil used in the experiment.

Cellulose Pressboard		Insulating Oil	
Brand	T_4_ pressboard	Brand	Karamay No.25 naphthenic mineral oil
Manufacturer	Taizhou Weidmann High Voltage Insulation Co. Ltd, Taizhou, China	Manufacturer	Chongqing Chuanrun Petroleum Chemical Co. Ltd., Chonngqing, China
Thickness	0.5 mm	*tanδ*	4 × 10^−4^
Tensile Strength	MD: 98 MPa, CMD: 47 MPa	Pour point	≤−45 °C
Density	0.96 g/cm^3^	Flash point	135 °C

**Table 2:** The detailed information of the tested pressboard.

DP = 1171	Group Number	1				
	Aging 0 Day	Moisture content (*mc%*)	0.91	2.10	2.87	4.07
		Label	P_11_	P_12_	P_13_	P_14_
DP = 854	Group Number	2				
	Aging 1 day	Moisture content (*mc%*)	1.17	2.47	3.24	4.07
		Label	P_21_	P_22_	P_23_	P_24_
DP = 674	Group Number	3				
	Aging 3 days	Moisture content (*mc%*)	1.12	2.02	3.11	4.15
		Label	P_31_	P_32_	P_33_	P_34_
DP = 424	Group Number	4				
	Aging 7 days	Moisture content (*mc%*)	1.18	2.32	3.39	4.17
		Label	P_41_	P_41_	P_43_	P_44_
DP = 279	Group Number	5				
	Aging 15 days	Moisture content (*mc%*)	1.28	2.31	3.35	4.47
		Label	P_51_	P_52_	P_53_	P_54_

**Table 3:** The integral factor values (*S*_1_, *S*_2_, and *S*_3_) of the imaginary part of the dielectric modulus.

DP = 1171	*mc%*			
	0.91	2.1	2.87	4.07
*S*_1_	3.45 × 10^−4^	4.16 × 10^−4^	3.50 × 10^−4^	6.24 × 10^−4^
*S*_2_	1.52 × 10^−3^	1.27 × 10^−2^	2.20 × 10^−2^	3.55 × 10^−2^
*S*_3_	1.05	1.31	1.89	3.86
DP = 854	*mc%*			
	1.17	2.47	3.24	4.07
*S*_1_	6.00 × 10^−4^	7.83 × 10^−4^	8.53 × 10^−4^	8.90 × 10^−4^
*S*_2_	3.65 × 10^−3^	1.55 × 10^−2^	2.84 × 10^−2^	4.32 × 10^−2^
*S*_3_	1.16	1.57	2.12	4.25
DP = 674	*mc%*			
	1.12	2.02	3.11	4.15
*S*_1_	8.35 × 10^−4^	1.09 × 10^−3^	1.10 × 10^−3^	1.21 × 10^−3^
*S*_2_	5.30 × 10^−3^	2.01 × 10^−2^	3.04 × 10^−2^	5.22 × 10^−2^
*S*_3_	1.10	1.75	3.23	7.55
DP = 424	*mc%*			
	1.18	2.32	3.39	4.17
*S*_1_	1.16 × 10^−3^	1.22 × 10^−3^	1.22 × 10^−3^	9.70 × 10^−4^
*S*_2_	8.93 × 10^−3^	2.46 × 10^−2^	3.36 × 10^−2^	5.72 × 10^−2^
*S*_3_	1.11	2.05	5.89	18.00
DP = 279	*mc%*			
	1.28	2.31	3.35	4.47
*S*_1_	1.41 × 10^−3^	1.22 × 10^−3^	1.02 × 10^−3^	1.62 × 10^−4^
*S*_2_	1.36 × 10^−2^	2.80 × 10^−2^	4.09 × 10^−2^	8.61 × 10^−2^
*S*_3_	1.20	2.86	7.17	25.23

**Table 4:** The values of *S*_4_ with various DPs and *mc%*.

**No**	**1**	**2**	**3**	**4**	**5**	**6**	**7**	**8**	**9**	**10**
DP	1171	1171	1171	1171	854	854	854	854	674	674
*mc*%	0.91	2.10	2.87	4.07	1.17	2.47	3.24	4.07	1.12	2.02
*S*_4_	0.06	3.50	11.00	38.00	0.15	5.80	19.00	330.00	0.39	7.80
**No**	**11**	**12**	**13**	**14**	**15**	**16**	**17**	**18**	**19**	**20**
DP	674	674	424	424	424	424	279	279	279	279
*mc*%	3.11	4.15	1.18	2.32	3.39	4.17	1.28	2.31	3.35	4.47
*S*_4_	25.00	530.00	0.56	9.70	31.00	770.00	0.72	17.00	56.00	1200

**Table 5:** The value of the *S_*1*_–S_*4*_* of 20 pressboards with different insulation conditions.

Aging Day(s)	DP	*mc%*	*S*_1_	*S*_2_	*S*_3_	*S*_4_	Target State
0	1171	0.91	3.45 × 10^−4^	1.52 × 10^−3^	1.05	0.06	1
		2.1	4.16 × 10^−4^	1.27 × 10^−2^	1.31	3.5	2
		2.87	3.50 × 10^−4^	2.20 × 10^−2^	1.89	11	3
		4.07	6.24 × 10^−4^	3.55 × 10^−2^	3.86	38	4
1	854	1.17	6.00 × 10^−4^	3.65 × 10^−3^	1.16	0.15	5
		2.47	7.83 × 10^−4^	1.55 × 10^−2^	1.57	5.8	6
		3.24	8.53 × 10^−4^	2.84 × 10^−2^	2.12	19	7
		4.07	8.90 × 10^−4^	4.32 × 10^−2^	4.25	330	8
3	674	1.12	8.35 × 10^−4^	5.30 × 10^−3^	1.10	0.39	9
		2.02	1.09 × 10^−3^	2.01 × 10^−2^	1.75	7.8	10
		3.11	1.10 × 10^−3^	3.04 × 10^−2^	3.23	25	11
		4.15	1.21 × 10^−3^	5.22 × 10^−2^	7.55	530	12
7	424	1.18	1.16 × 10^−3^	8.93 × 10^−3^	1.11	0.56	13
		2.32	1.22 × 10^−3^	2.46 × 10^−2^	2.05	9.7	14
		3.39	1.22 × 10^−3^	3.36 × 10^−2^	5.89	31	15
		4.17	9.70 × 10^−4^	5.72 × 10^−2^	18.00	770	16
15	279	1.28	1.41 × 10^−3^	1.36 × 10^−2^	1.20	0.72	17
		2.31	1.22 × 10^−3^	2.80 × 10^−2^	2.86	17	18
		3.35	1.02 × 10^−3^	4.09 × 10^−2^	7.17	56	19
		4.47	1.62 × 10^−4^	8.61 × 10^−2^	25.23	1200	20

**Table 6:** Classification of transformer cellulose insulation conditions.

Aging Condition	Damp Condition	Conditions Number
DP = 900−1400	*mc*% = 0%−1.5%, Dry	T_1_
	*mc*% = 1.5%−3%, Slight damp	T_2_
	*mc*% = 3%−4%, Damp	T_3_
	*mc*% > 4%, Serious damp	T_4_
DP = 700−900	*mc*% = 0%−1.5%, Dry	T_5_
	*mc*% = 1.5%−3%, Slight damp	T_6_
	*mc*% = 3%−4%, Damp	T_7_
	*mc*% > 4%, Serious damp	T_8_
DP = 500−700	*mc*% = 0%−1.5%, Dry	T_9_
	*mc*% = 1.5%−3%, Slight damp	T_10_
	*mc*% = 3%−4%, Damp	T_11_
	*mc*% > 4%, Serious damp	T_12_
DP = 300−500	*mc*% = 0%−1.5%, Dry	T_13_
	*mc*% = 1.5%−3%, Slight damp	T_14_
	*mc*% = 3%−4%, Damp	T_15_
	*mc*% > 4%, Serious damp	T_16_
DP < 300	*mc*% = 0%−1.5%, Dry	T_17_
	*mc*% = 1.5%−3%, Slight damp	T_18_
	*mc*% = 3%−4%, Damp	T_19_
	*mc*% > 4%, Serious damp	T_20_

**Table 7:** Relevant parameters of the newly prepared samples.

Sample	*DP*	*mc%*	*S*_1_	*S*_2_	*S*_3_	*S*_4_
*NS*_1_	726	2.41	9.38 × 10^−4^	3.52 × 10^−2^	3.89	160
*NS*_2_	313	1.21	1.06 × 10^−3^	6.7 × 10^−3^	1.16	0.46
*NS*_3_	293	1.28	1.26 × 10^−3^	1.00 × 10^−2^	1.11	1.10

**Table 8:** Recognition results of new samples.

Sample	Predictive Results (Conditions Number)	Predictive DP	Practical DP	Predictive *mc%*	Practical *mc%*
*NS*_1_	T_8_	700−900	726	>4%	2.41
*NS*_2_	T_13_	300−500	313	0%−1.5%	1.21
*NS*_3_	T_17_	<300	293	0%−1.5%	1.28

**Table 9:** Prediction results of grey relational analysis.

Sample	Predictive Results (Conditions Number)	Predictive DP	Practical DP	Predictive *mc%*	Practical mc%
*NS*_1_	T_4_	900−1400	726	>4%	2.41
*NS*_2_	T_17_	<300	313	0%−1.5%	1.21
*NS*_3_	T_13_	300−500	293	0%−1.5%	1.28
